# Alloying effect of Ni-Mo catalyst in hydrogenation of N-ethylcarbazole for hydrogen storage

**DOI:** 10.3389/fchem.2022.1081319

**Published:** 2022-12-13

**Authors:** Bin Wang, Qian Dong, Si-Yao Wang, Pei-Ya Li, Shi-Yuan Wang, Shu-Han Lu, Tao Fang

**Affiliations:** ^1^ Shaanxi Key Laboratory of Energy Chemical Process Intensification, School of Chemical Engineering and Technology, Xi’an Jiaotong University, Xi’an, China; ^2^ Engineering Research Center of New Energy System Engineering and Equipment, University of Shaanxi Province, Xi’an, Shaanxi, China

**Keywords:** LOHCs, N-ethylcarbazole, Ni-based catalyst, hydrogenation, alloying effect

## Abstract

Liquid organic hydrogen storage with N-ethylcarbazole (NEC) as a carrier is a very promising method. The use of precious metal hydrogenation catalysts restricts the development in industrial grade. Efficient and low-cost hydrogen storage catalysts are essential for its application. In this work, a Ni-Mo alloy catalyst supported by commercial activated carbon was synthesized by impregnation method, and the Ni-Mo ratio and preparation conditions were optimized. The catalyst was characterized by XRD, XPS, H_2_-TPR, SEM, and TEM. The results showed that the doping of Mo could dramatically promote the catalytic hydrogenation of N-ethylcarbazole by the Ni-based catalyst. More than 5.75 wt% hydrogenation could be achieved in 4 h using the Ni-Mo catalyst, and the selectivity of the fully hydrogenated product 12H-NEC could be effectively improved. This result reduces the cost of hydrogenation catalysts by more than 90% and makes liquid organic hydrogen storage a scaled possibility.

## 1 Introduction

Hydrogen energy is considered to be the ultimate clean energy solution (Crabtree, 2017). Due to its low density, high diffusion with flammability and explosiveness, the large-scale use of hydrogen is limited by the way it is stored and transported (Grubel et al., 2020). At the present stage, the commercial hydrogen storage methods are mainly high-pressure gaseous hydrogen and low-temperature liquefied hydrogen, and yet they still cannot meet the requirements of large-scale and long-distance transportation considering the process complexity, process energy loss and safety factor (Muller, 2019). Researchers have developed a series of hydrogen carriers, such as metal hydride, liquid organic hydrogen carriers (LOHCs), modified activated carbon and glass microspheres, to fulfill industrial applications (Ren et al., 2017; Abdalla et al., 2018). Among these candidates, LOHCs, which have attracted widespread attention because of their high storage density, high safety, and compatibility with existing liquid fuel infrastructure, are the optimum selection for large-scale and medium/long-distance transportation of hydrogen (Preuster et al., 2017; He et al., 2019; Niermann et al., 2019). It achieves reversible reactions of hydrogenation and dehydrogenation in unsaturated bonds with the help of specific catalysts.

The representative LOHCs are toluene, naphthalene, dibenzyltoluene (DBT), and N-ethylcarbazole (NEC) (Jorschick et al., 2017; Nakaya et al., 2020; Tuo et al., 2021; Xue et al., 2021). In 2004, aromatic heterocyclic organic compounds were first proposed as hydrogen storage media (Pez et al., 2008). Clot et al. (2007) found that the introduction of heteroatoms such as N and O in aromatic heterocyclic compounds facilitates the dehydrogenation reaction by density functional theory (DFT). Among the LOHCs, NEC is the most favored. It is the first proposed LOHC that can achieve hydrogenation/dehydrogenation cycles below 200°C. It has low volatility and toxicity, and its theoretical hydrogen storage density can reach 5.79 wt% with a released hydrogen purity of 99.99% (Tcichmann et al., 2012).

Efficient and economical hydrogen storage catalysts are essential for the industrial application of NEC. According to the literature studies, loaded precious metal catalysts have been widely used, including ruthenium-based catalysts for hydrogenation and palladium-based catalysts for dehydrogenation (Wang et al., 2017; Wu et al., 2019a; Wang et al., 2019; Yu et al., 2020). The key challenge limiting the use of non-precious metal catalysts is the slow reaction kinetics and low product selectivity. To reduce the feedstock cost while maintaining an activity similar to precious metal catalysts, researchers have started to turn their attention to tuning the structure of non-precious metal catalysts, of which nickel-based catalysts are the main ones owing to their certain activity. The main attempts are reducing the amount of precious metals by doping with nickel, synthesizing nickel-containing alloy catalysts, and modifying the catalyst supports. The same doping method was used in the research of Yu et al. (2020), and they also focused on the hydrogenation activity caused by the crystal form of TiO_2_. In contrast, Liu et al. (2021) prepared high-performance Rh-Ni/γ-Al_2_O_3_ catalysts by doping very small amounts of Rh in Ni. Ding et al. (2021) prepared Ni_70_/AlSiO-1/1 catalysts by using a new carrier AlSiO-1, which could weaken the metal-carrier interaction and reduce the formation of NiAl_2_O_4_ species to facilitate the reduction of Ni. Wu et al. (2019b) prepared catalysts by combining Ni/Al_2_O_3_ with a metal hydrogen storage material of YH_3_ as a catalyst, which significantly improved the hydrogenation process of NEC. Yu et al. (2021) demonstrated that the hydrogen storage alloy LaNi_5.5_ can be directly used for reversible hydrogenation and dehydrogenation of NEC with high efficiency and cycle stability. The properties of these catalysts are shown in Table 1.

**TABLE 1:** Catalytic performance of nickel-containing hydrogenation catalysts reported.

Catalysts	The mass ratio of catalyst to NEC	Diluting	Conditions	Time/h	H_2_ update/wt%	References
5 wt% Ru-Ni/TiO_2_	.05	No	150°C, 7 MPa	24	5.65	Yu et al. (2020)
.1 wt%Rh-15wt% Ni/γ-Al_2_O_3_	.05	No	160°C, 6 MPa	2	5.63	Liu et al. (2021)
70 wt% Ni/AlSiO-1/1	.1	40 ml N-hexane	150°C, 7 MPa	1.5	>5.75	Ding et al. (2021)
5 wt% Ni/Al_2_O_3_	.125	No	180°C, 10 MPa	1.5	>5.75	Wu et al. (2019b)
+4 times YH_3_ to catalyst		No	150°C, 3 MPa	4.5	>5.75	
Pure LaNi_5.5_	.1	No	180°C, 7 MPa	8	5.63	Yu et al. (2021)

In this work, a Ni_4_Mo/AC bimetallic catalyst was synthesized and used for the hydrogenation reaction of NEC, which can accomplish efficient and highly selective hydrogenation without using precious metals. The catalyst characterization revealed that Ni_4_Mo/AC formed an alloy structure in the preparation process. In addition, Ni_4_Mo/AC can reduce the dissociation energy of hydrogen and thus improve the catalytic hydrogenation activity. This work can provide a reference for the design and synthesis of non-precious metal NEC hydrogenation catalysts with high activity and high selectivity.

## 2 Materials and methods

### 2.1 Characterization methods

X-ray diffraction (XRD, Bruker D8) analysis was performed to determine the crystalline phase of the samples. Hydrogen temperature programmed reduction (H_2_-TPR, AutoChem1 II 2920) analysis was performed with a thermal conductivity detector (TCD) to study the reduction behavior of the catalyst precursor. In H_2_-TPR, the prepared samples were pretreated by a programmed ramp-up at 10°C/min to 300°C dry under 30 ml/min Ar inert gas protection, kept purged for 1 h and then cooled to room temperature, followed by heating from 80°C to 800°C at 10°C/min in a 10% H_2_/Ar mixture at 30 ml/min, and the amount of H_2_ consumed was characterized by a thermal conductivity detector (TCD). X-ray photoelectron spectroscopy (XPS, Thermo Scientific K-Alpha) was performed to analyze the metallic elements. The surface morphology and metal distribution of the catalysts were obtained using scanning electron microscopy (SEM, Zeiss MERLIN Compact). The metal particle size of the catalyst was obtained using transmission electron microscopy (TEM, JEM 2100F).

### 2.2 Preparation of catalysts

A simple impregnation method was used to prepare NiMo/AC catalysts. First, 1 g of activated carbon (AC) was dispersed in 10 ml of deionized water, and nickel chloride hexahydrate and ammonium molybdate tetrahydrate were added at a total mass fraction of 15% and stirred for 24 h. Then, the pH value was adjusted to eight and stirred for 3 h. The mixture was washed 3–5 times using a centrifuge to ensure that the supernatant was free of chloride ions. The cleaned samples were dried in a vacuum drying oven at 60°C. The samples were homogeneously ground with an agate mortar and then heated in a tube furnace at 10% H_2_/N_2_ for 3 h at a certain temperature to obtain the NiMo/AC catalyst.

### 2.3 Hydrogen uptake experiment

In a 100 ml high-pressure reactor, 2 g of NEC, 18 ml of decalin and 0.4 g of catalyst were added. Argon was used to displace the gas in the reactor. After heating to 150°C, hydrogen gas was introduced to pressure the reactor to 8 MPa. The temperature and pressure data of the reactor were recorded by sensors on a computer. The reaction was stopped after 4 h regardless of the extent of the reaction. After the reactor was cooled to room temperature, the product was collected to measure the composition using gas chromatography, and then the amount of hydrogen uptake was identified.

## 3 Results and discussion

Eight different nickel-molybdenum catalysts were prepared using conventional impregnation methods and then subjected to systematic physicochemical characterization and NEC hydrogenation reaction tests to investigate the effects of different nickel-molybdenum ratios and different reduction temperatures on the catalytic performance.

### 3.1 Catalyst characterization of the Ni_4_Mo/AC catalyst

**FIGURE 1:**
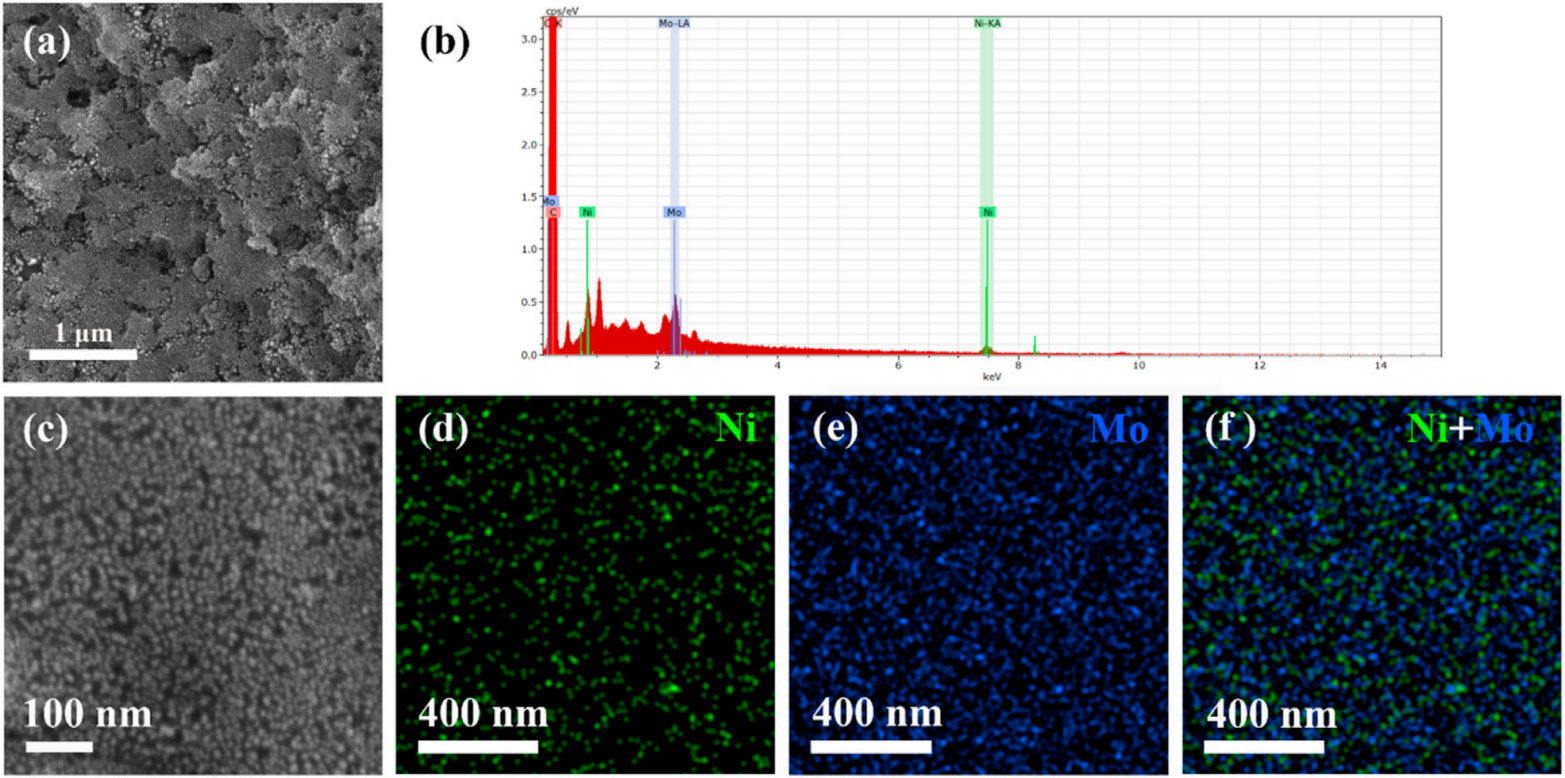
Structural characterization of Ni_4_Mo/AC. **(A,C)** SEM image. **(B)** EDS spectrum. **(D–F)** EDS elemental mapping of Ni and Mo in Ni_4_Mo/AC.

The chemical composition and elemental profiles of the catalysts were obtained using SEM in combination with an EDS analyzer. The SEM images in Figures 1A, C show some morphological features of the Ni_4_Mo/AC catalyst. The surface of the material was rough, which was a common feature observed in the SEM images of the carbon support. The EDS spectrum in Figure 1B shows the composition of the prepared materials. In the sample, C, Ni, and Mo were found to be present in large amounts. Both Ni and Mo had double peaks, and C had only a single peak, showing the relative amounts of these elements. C has the most intense peak, as it was the main component of the carrier (Naushad et al., 2019). The different colors in the mapping image (Figures 1D–F) of Ni_4_Mo/AC showed the distribution of Ni and Mo in the prepared catalyst. The distribution revealed that Ni and Mo were homogeneously dispersed in different regions of the carbon-supported surface without obvious agglomeration or segregation. Such a uniform distribution also implied that an alloy of NiMo was formed (Wang et al., 2013). These better morphologies are rare in Ni-based catalysts made by gas reduction (Shanmuganandam and Ramanan, 2016; Wu et al., 2019b). In our preparation, proper metal loading based on the support and stable conditions contributed to this result together.

**FIGURE 2:**
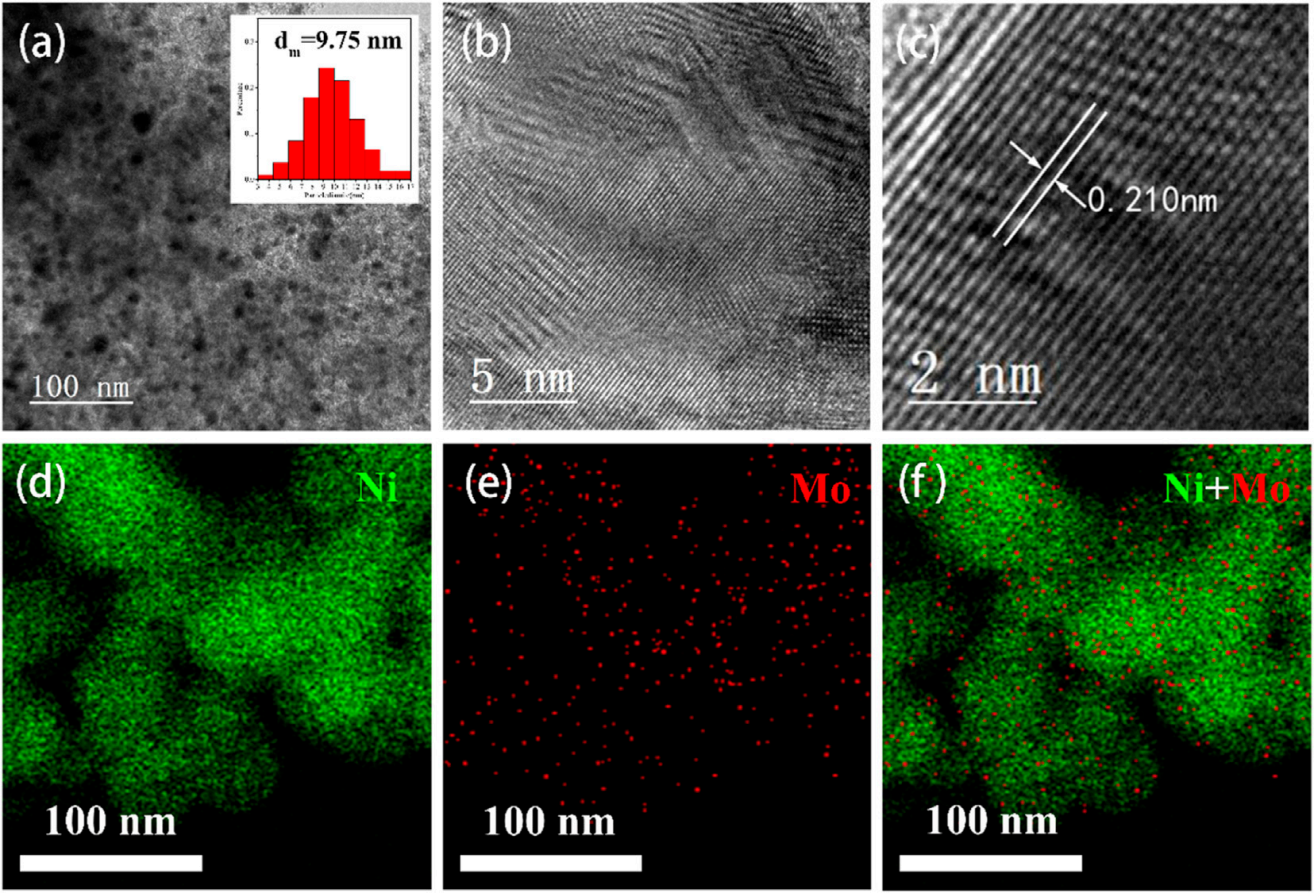
Structural characterization of Ni_4_Mo/AC. **(A–C)** TEM image. **(D–F)** EDS elemental mapping of Ni and Mo in Ni_4_Mo/AC.

To examine the crystal morphology and structure, the prepared Ni_4_Mo/AC samples were studied by TEM. Figures 2A, B shows TEM micrographs of the Ni_4_Mo/AC catalyst. The images illustrate the particle size and metal dispersion of Ni_4_Mo/AC. It is obvious that the metal particles of Ni_4_Mo/AC exhibited high homogeneity, as shown in the SEM image of Figure 1B. The statistical analysis of particle size is given as an inset in Figure 2A, showing that for the Ni_4_Mo/AC catalyst, the average particle size was approximately 9.75 nm within a narrow range. In addition, the high-resolution image (Figure 2C) showed a lattice spacing of 0.210 nm, which was consistent with 0.210 nm for the Ni_4_Mo (111) facet (Wang et al., 2021). This indicated the formation of an ordered Ni_4_Mo nickel-molybdenum alloy in the catalyst. The mapping image (Figures 2D-F) further demonstrated the uniform dispersion of Ni and Mo on the carrier surface and the formation of an alloy structure.

**FIGURE 3:**
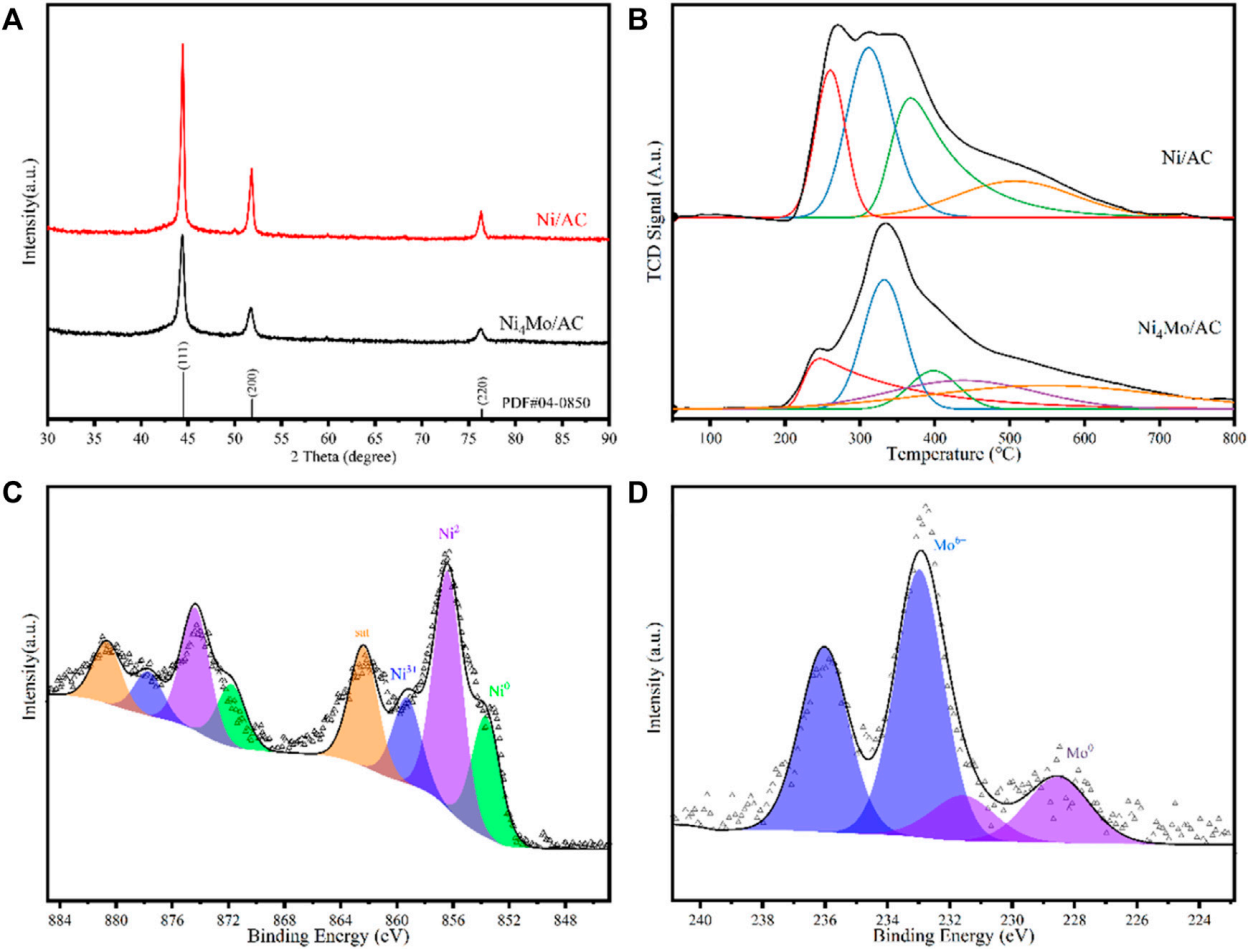
**(A)** XRD pattern of Ni_4_Mo/AC. **(B)** H_2_-TPR of samples before reduction. **(C)** High-resolution XPS spectrum of Ni 2p in Ni_4_Mo/AC. **(D)** High-resolution XPS spectrum of Mo 3d in Ni_4_Mo/AC.

The powder XRD pattern of Ni_4_Mo/AC is shown in Figure 3A. The diffraction peaks at 2θ values of 44.398°, 51.704°, and 76.264° corresponded to the (111), (200), and (220) facets of the Ni_4_Mo alloy, respectively. The diffraction peak of Ni_4_Mo was shifted to a small angle compared to the standard card of Ni (PDF#04-0850), which was due to the doping of molybdenum with a larger atomic weight than that of Ni. In addition, comparing the XRD patterns of Ni_4_Mo/AC and Ni/AC (Figure 3A), it could be seen that the half-peak width becomes larger, showing that the doping of a certain amount of Mo reduced the crystallinity of the pure nickel phase. These results indicated that an ordered Ni_4_Mo alloy was formed on the activated carbon after doping with a small amount of molybdenum.

Temperature-programmed reduction (TPR) was used to study the interactions between the active components of the loaded catalysts. To evaluate the interactions between nickel and molybdenum, Ni_4_Mo/AC, Ni/AC and Mo/AC samples before reduction were subjected to H_2_-TPR (Figure 3B). Under the same conditions, all metals could be reduced. For Mo/AC (Supplementary Figure S1), two major peaks were observed, one at 360°C and the other at 470°C, as an inverse peak, which was due to the reaction of the sample to form MoC during the temperature-programmed reduction (Qu et al., 2003). For Ni/AC, the maximum reduction temperature showed a broad band centered at 250°C and 350°C. In contrast, the H_2_-TPR pattern for Ni_4_Mo/AC could be observed with a peak at 340°C and a lap-shoulder peak at 250°C. The H_2_-TPR of Ni/AC and Ni_4_Mo/AC samples before reduction were analyzed after peak fitting. In the former sample, the reduction peaks at 260°C, 296°C, and 344°C were attributed to the progressive reduction of metallic nickel, and the reduction peak at 485°C was attributed to the reduction of surface and bulk oxygen in activated carbon (Le et al., 2017). In the latter samples, the reduction peaks at 224°C, 332°C, and 397°C were attributed to the reduction of metallic nickel, and the reduction peak at 437°C was attributed to the reduction of metallic molybdenum. The above split-peak results indicated that the reduction process of nickel is changed after doping with molybdenum metal. These results suggested that the mixing of Ni-Mo metal differs from the reduction process of elemental Ni overlying elemental Mo and that there was an interaction between the two metals, meaning that a new alloy was formed.

XPS was used to study the detailed information on the distribution of elements Ni and Mo. The XPS spectra (Supplementary Figure S2) confirmed that only the expected elements Ni, Mo, C, and O from metal oxidation were present in the Ni_4_Mo/AC catalyst. The corresponding Ni2p spectra and Mo3d spectra of Ni_4_Mo/AC are shown in Figures 3C, D, where 853.66, 856.37, 860.13, and 862.38 eV corresponded to Ni, Ni^2+^, Ni^3+^ and satellite peaks, 228.53 and 231.58 eV corresponded to Mo, and 232.97 and 236.02 eV corresponded to Mo^6+^ (Xiao et al., 2021). The possible reason for the presence of Ni and Mo oxides was the oxidation caused by the catalyst transfer process, in which it is easy to form an oxide layer on the surface owing to the active metal properties of nanosized Ni and Mo.

### 3.2 Catalytic performances

**FIGURE 4:**
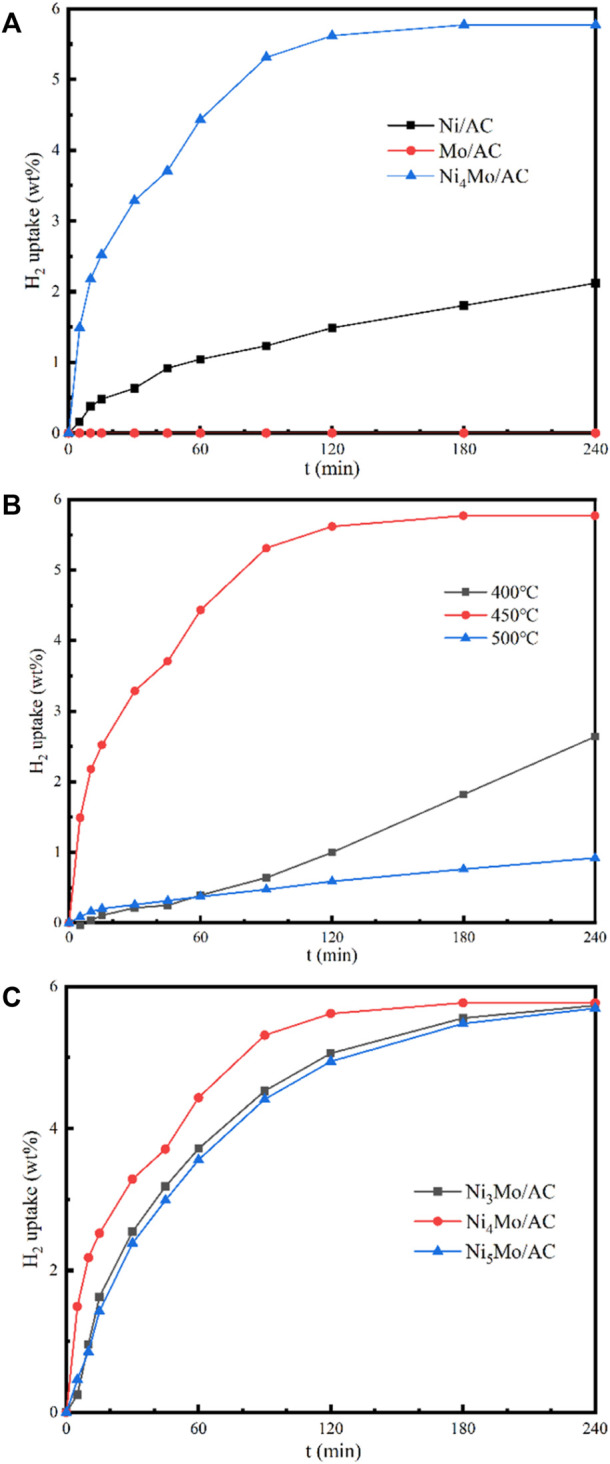
Hydrogenation kinetics of different catalysts. **(A)** Different loading metals; **(B)** Different reduction temperatures; **(C)** Different Ni/Mo ratios.

The catalytic performance of the prepared catalysts is shown in Figure 4 and Table 2. In Figure 4A, it can be seen that Mo/AC has no hydrogenation performance. Ni/AC had a lower hydrogenation activity with only 58.46% conversion and 23.6% selectivity for the final product at 4 h. In contrast, Ni_4_Mo/AC had a high catalytic activity, and finally, the 4-h conversion reached 100%. The final product selectivity was 98.73%, and the hydrogen uptake amount reached 5.77 wt%. This indicated that the hydrogenation performance of the catalyst was greatly improved after doping with a certain amount of Mo, which has no activity in hydrogenation. As we mentioned above, the catalyst formed a Ni_4_Mo alloy, which could significantly reduce the dissociation energy of hydrogen (Wang et al., 2021). This resulted in an enhancement of the contact within active hydrogen atoms and LOHC molecules, thus promoting the formation of C-H bonds in the N-heterocycles.

**TABLE 2:** Hydrogenation activities of different catalysts.

Catalysts	12H-NEC/%	8H-NEC/%	4H-NEC/%	NEC/%	H_2_ uptake/wt%
Ni/AC	23.6	4.04	30.82	41.54	2.12
Mo/AC	.00	.00	.00	100.00	.00
Ni_3_Mo/AC	97.11	2.89	.00	0.00	5.73
Ni_4_Mo/AC-400°C	24.16	13.07	38.25	24.52	2.64
Ni_4_Mo/AC-450°C	98.73	1.27	.00	.00	5.77
Ni_4_Mo/AC-500°C	7.37	1.60	22.53	68.50	.92
Ni_5_Mo/AC	94.96	5.04	.00	.00	5.69

**FIGURE 5:**
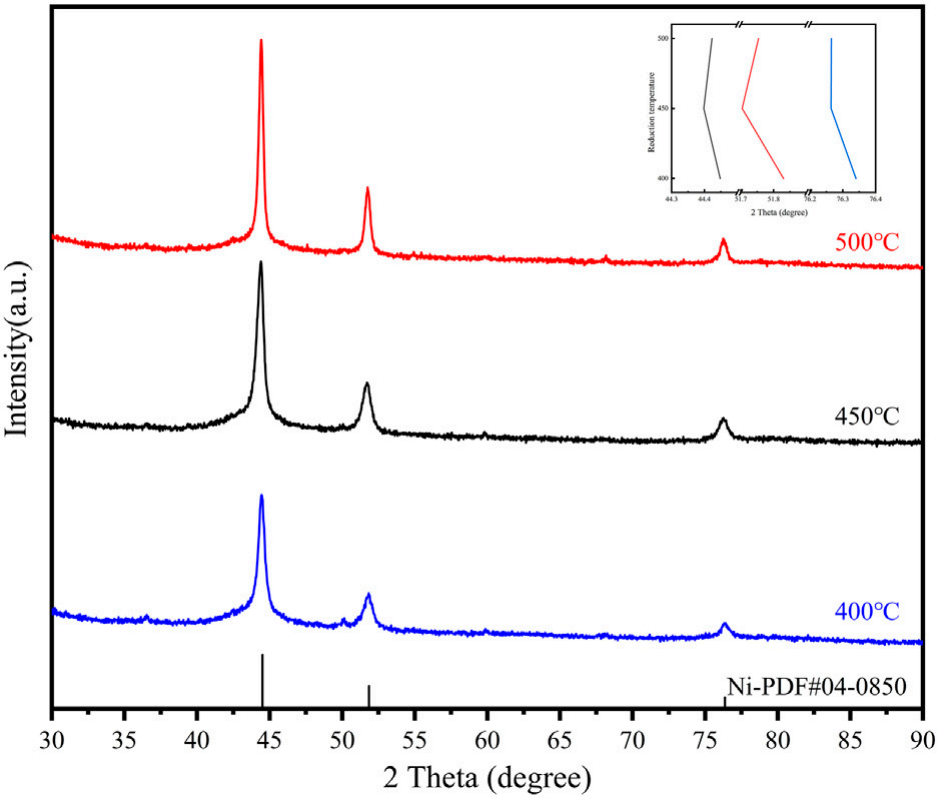
XRD patterns of Ni_4_Mo/AC with different reduction temperatures. The inset image shows the trend in the shift of the three main peaks.

**FIGURE 6:**
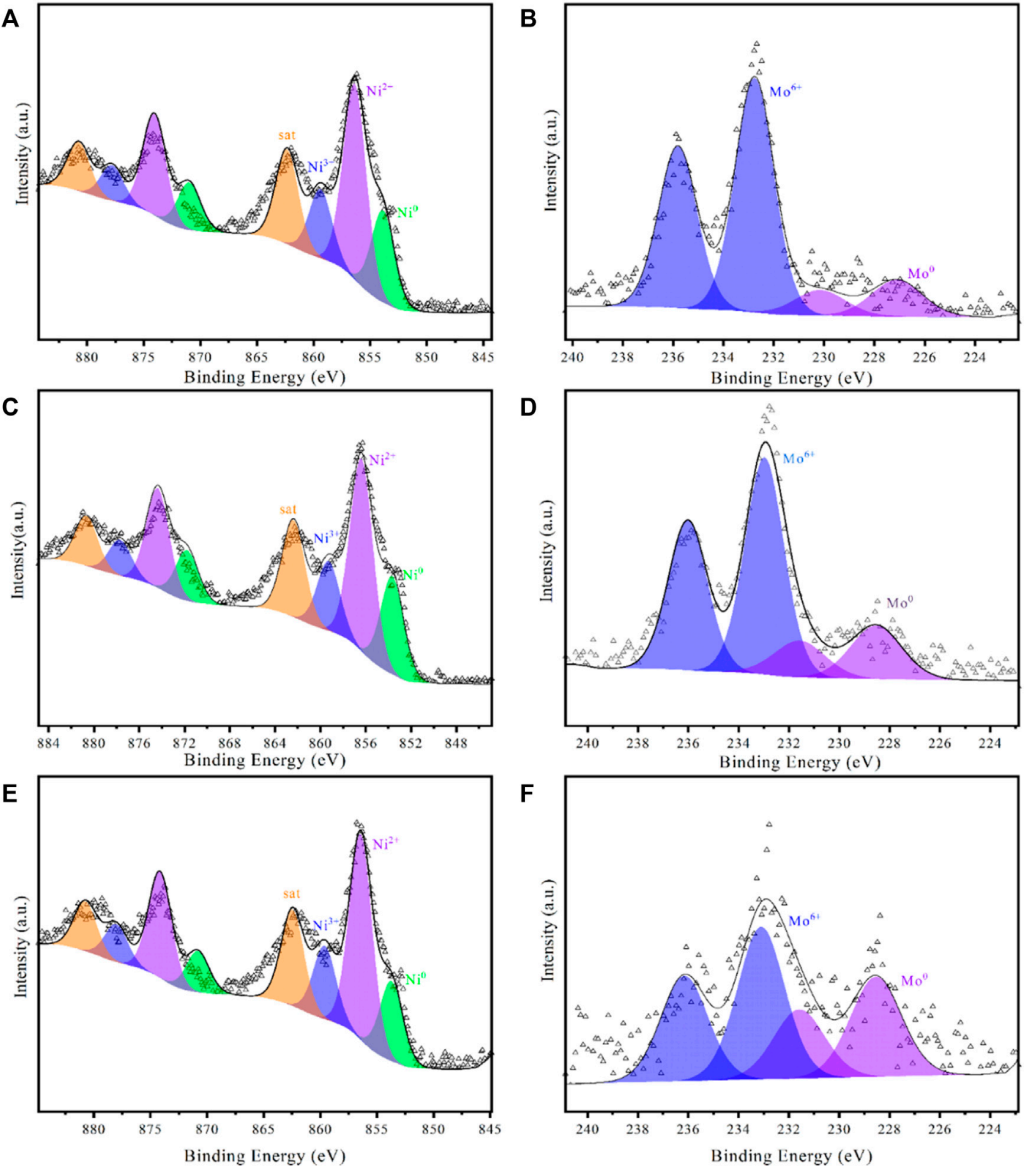
XPS patterns of Ni_4_Mo/AC with different reduction temperatures. **(A,B)** 400°C; **(C,D)** 450°C; **(E,F)** 500°C.

**FIGURE 7:**
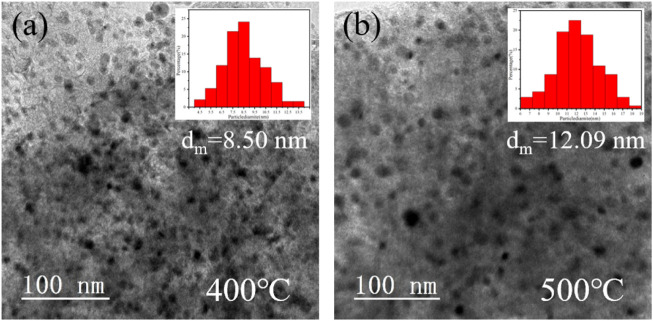
TEM images and particle size distributions of samples with different reduction temperatures **(A)** 400°C, **(B)** 500°C.

The reduction temperature of the loaded catalyst affects the morphology and particle size, which in turn affects the catalytic performance. The catalyst precursors were reduced for comparison at 400°C, 450°C, and 500°C, and a series of characterizations were performed. The three catalysts were also tested for the hydrogenation of NEC (Figure 4B). It can be realized that the effect of temperature is non-linear. The catalytic performance was best at a reduction temperature of 450°C, followed by 400°C and 500°C. Although the reduction temperature only differs by 50°C, the reactivity more than doubles. The activity of the catalyst prepared at 500°C is not as good as that of Ni/AC, while the catalyst prepared at 400°C is only similar to that of Ni/AC. Characterizations were carried out to analyze the cause of this phenomenon. XRD patterns (Figure 5) showed that the diffraction peaks of Ni were most significantly shifted to a small angle at a reduction temperature of 450°C, which indicated the best doping effect of molybdenum. The peaks in Ni_4_Mo/AC-400°C did not exhibit a significant shift. The XPS patterns (Figure 6) of the three catalysts showed a slightly higher Ni content in the metallic state at a reduction temperature of 450°C. However, the valence distribution of Mo is more significantly affected by temperature, which directly reflects the alloying degree of NiMo. Compared to Ni/AC, it can be claimed from the XPS fitting data (Table 3) that Ni_4_Mo/AC had the highest alloying at 450°C. The excessively high 0-valent Mo content at 500°C indicates that this temperature may be beneficial to the *in situ* reduction of metallic Mo, making the segregation of Mo more obvious. This can also be corroborated by its poorer activity, which is closer to that of Mo/AC. The low 0-valent Mo content at 400°C indicates that the reduction process of the catalysts was not carried out thoroughly enough. The reduction rate of Mo at lower temperatures could not match the formation of Ni, so better alloying could not be formed, which can also be confirmed from the H_2_-TPR of Mo/AC. In addition, TEM (Figure 7) of the three catalysts showed that the particle size of the catalysts gradually increased with increasing temperature, but the larger particle size did not favor the catalytic hydrogenation process of NEC (Yu et al., 2020). At 500°C, the higher reduction temperature led to a certain extent of aggregation of metals. The active sites are thus reduced, which also affects reactivity. Therefore, an appropriate reduction temperature of 450°C is an important condition to ensure catalyst activity by affecting alloying.

**TABLE 3:** XPS data of samples.

Sample name	Reduction temperature/°C	Atomic percentage					
		Ni	Ni^2+^	Ni^3+^	sat	Mo	Mo^6+^
Ni/AC	450	20.96	43.96	21.66	13.42	—	—
Ni_3_Mo/AC	450	21.94	42.45	15.1	20.51	25.64	74.36
Ni_4_Mo/AC	450	21.26	42.19	15.28	21.26	23.33	76.67
Ni_5_Mo/AC	450	19.28	43.79	15.69	21.24	32.14	67.86
Ni_4_Mo/AC	400	20.47	43.26	15.28	20.98	16.67	83.33
Ni_4_Mo/AC	500	17.73	45	15.91	21.36	41.18	58.82

**FIGURE 8:**
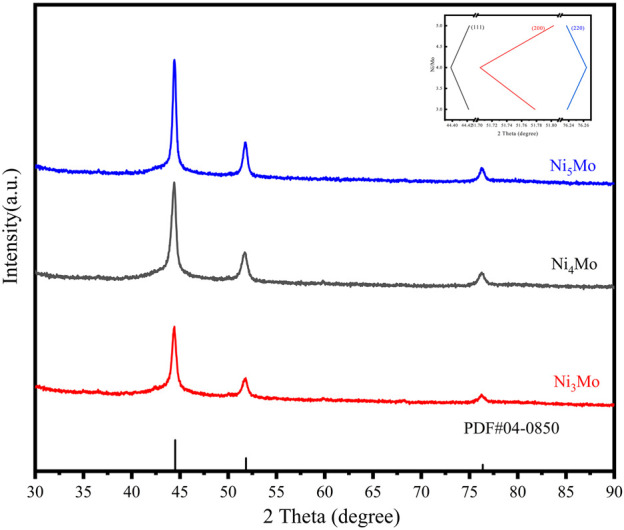
XRD patterns of samples with different ratios of Ni to Mo. The inset image shows the trend in the shift of the three main peaks.

It was found that NiMo/AC catalysts with different Ni-Mo ratios also had different catalytic effects. Three catalysts with different Ni-Mo ratios, Ni_3_Mo/AC, Ni_4_Mo/AC, and Ni_5_Mo/AC, were prepared, and they were subjected to characterization and NEC hydrogenation tests. The hydrogenation curves of the three catalysts are shown in Figure 4C. The best catalytic effect of Ni_4_Mo/AC and the worst catalytic effect of Ni_5_Mo/AC were shown, while Ni_3_Mo/AC was slightly more active than Ni_5_Mo/AC. Similar to what we mentioned above, this could also be attributed to the alloying degree of nickel and molybdenum at different ratios. In the XRD patterns of all three catalysts (Figure 8), distinct nickel diffraction peaks could be observed, but the nickel diffraction peaks in Ni_4_Mo/AC were most clearly shifted to a small angle, indicating that the best alloying effect was achieved at a Ni-Mo ratio of 4, followed by Ni_3_Mo/AC and then Ni_5_Mo/AC. The XPS patterns (Supplementary Figure S4) of the three catalysts showed approximately the same peak splitting results, and the difference also came from the valence distribution of Mo under different NiMo ratios. The reactivity is linearly related to the content of 0-valence Mo, that is, the degree of possible segregation. In addition, H_2_-TPR tests were performed on the prepared samples before reduction. The H_2_-TPR patterns (Supplementary Figure S5) of the three catalysts have similar shapes, but the highest reduction peaks were found for Ni_4_Mo/AC, which may be responsible for the high alloying of the catalysts. It can be realized that the difference in alloying degree caused by the ratio change is not as significant as the effect of temperature, so the catalyst activity changes little by the ratio.

## 4 Conclusion

N-ethylcarbazole is considered the most promising liquid organic hydrogen carrier. However, the high cost caused by precious metal catalysts in the hydrogenation process limits their development to industrial grade. In this work, Ni_4_Mo/AC bimetallic catalysts were prepared by the impregnation method and used in the hydrogenation reaction of NEC to reduce the cost of hydrogenation catalysts, and the selectivity of the final product 12H-NEC could be improved. By maintaining high conversion and high selectivity, Ni_4_Mo/AC has the lowest metal content, even among the reported non-precious metal catalysts. Its manufacturing cost is more than 90% lower than that of precious metals and can guarantee similar activity. The conversion can reach 100% in 4 h at 150°C and 8 MPa, and the selectivity of the final hydrogenated product can be 98.73% with 5.77% hydrogenation. This performance attributed to the Ni_4_Mo alloy can reduce the dissociation energy of hydrogen. Combined with XRD, TEM, SEM, H_2_-TPR, and XPS analyses, it was found that increasing the alloying degree and decreasing the metal particle size of the catalysts could improve the hydrogenation activity and selectivity, which was affected by the reduction temperature and metal ratio of the catalysts.

## Data Availability

The raw data supporting the conclusion of this article will be made available by the authors, without undue reservation.
