# Supplementary material for: Alloying effect of Ni-Mo catalyst in hydrogenation of N-ethylcarbazole for hydrogen storage
Source: Front Chem. 2022 Dec 13;10:1081319. doi: 10.3389/fchem.2022.1081319 (PMC9792484; doi:10.3389/fchem.2022.1081319)
Supplement: Supplementary file 1 [file DataSheet1.docx]

Supporting Information of

Alloying effect of Ni-Mo catalyst in hydrogenation of N-ethylcarbazole for hydrogen storage


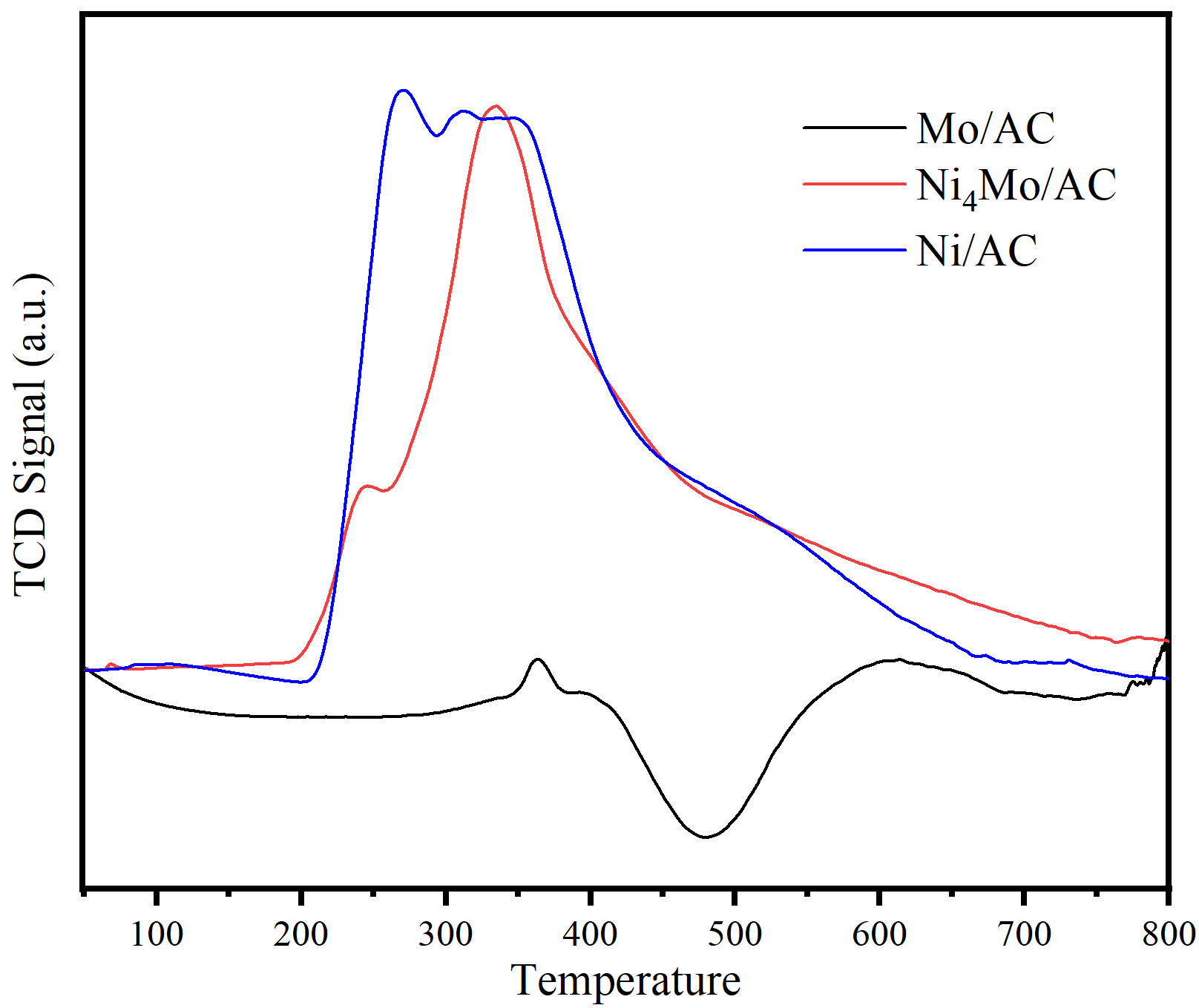


**Figure S1.** H_2_-TPR profiles of Mo/C, Ni_4_Mo/AC and Ni/AC.


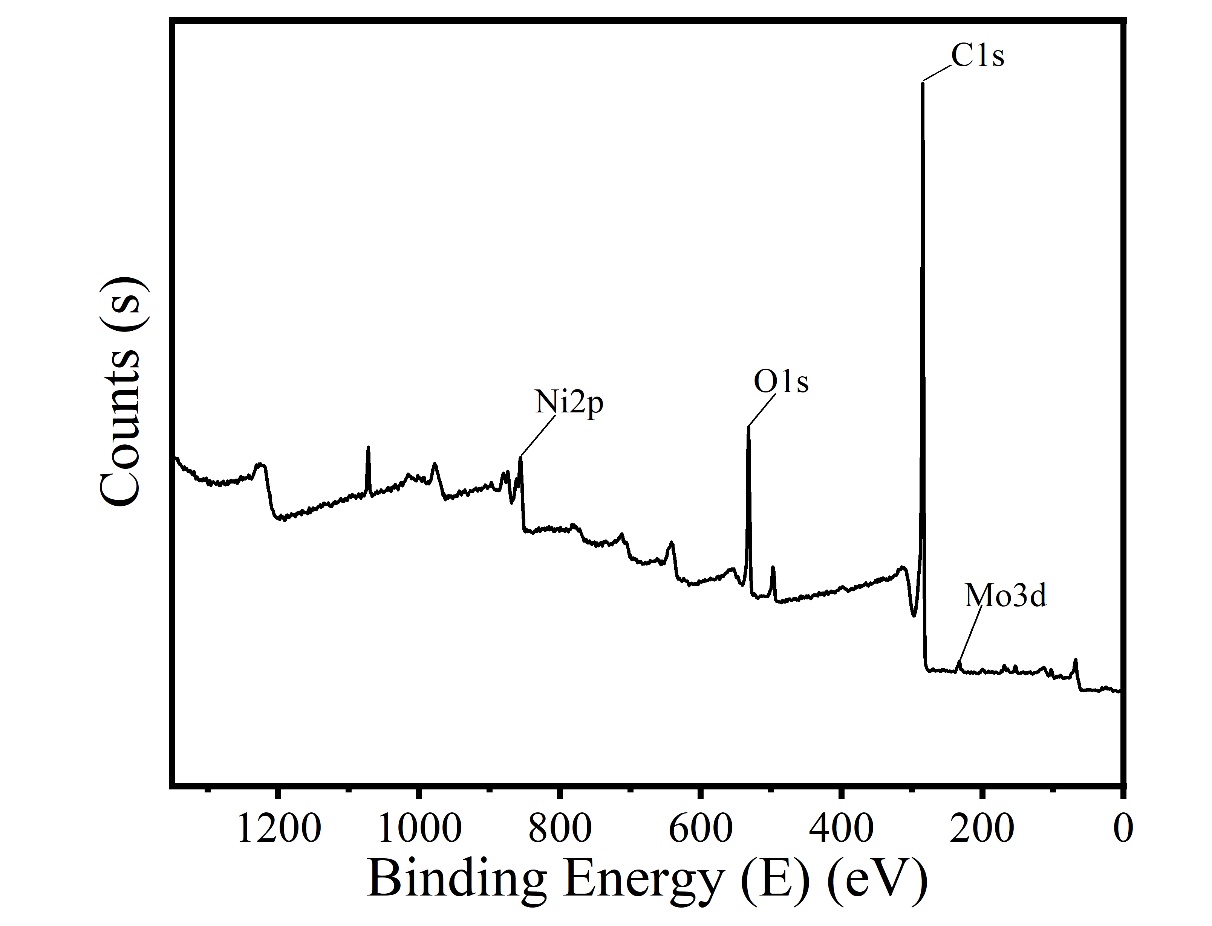


**Figure S2.** XPS spectra of Ni_4_Mo/AC.

**Figure S3.** XPS patterns of Ni/AC.


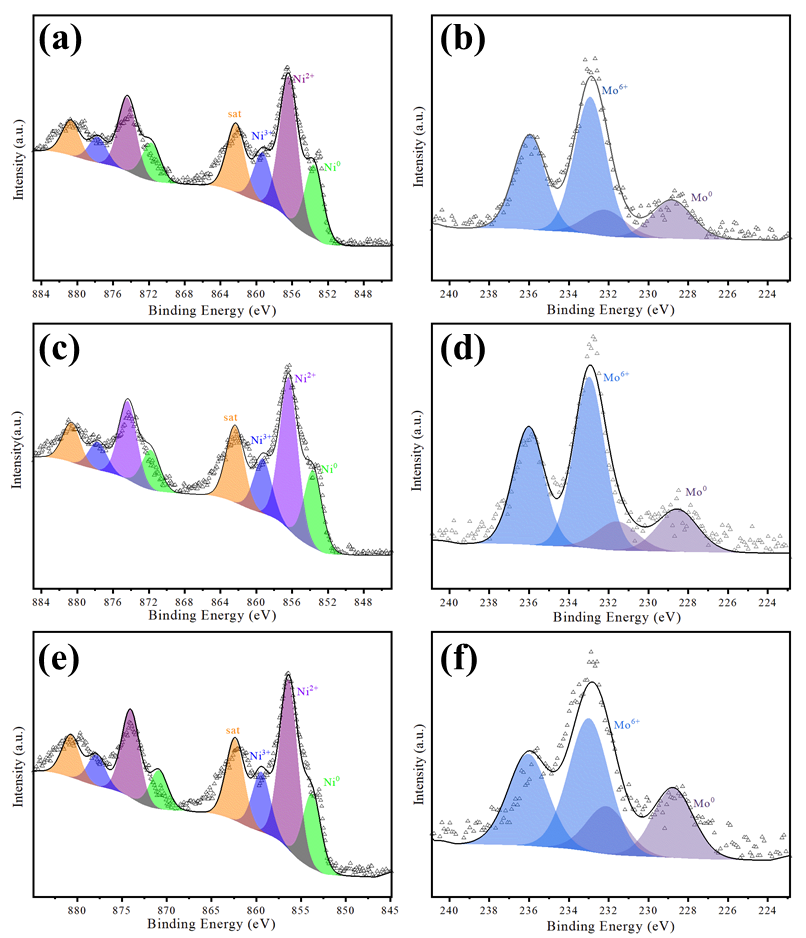


**Figure S4.** XPS patterns of samples with different ratio of Ni to Mo.

(a, b) Ni_3_Mo/AC; (c, d) Ni_4_Mo/AC; (e, f) Ni_5_Mo/AC.


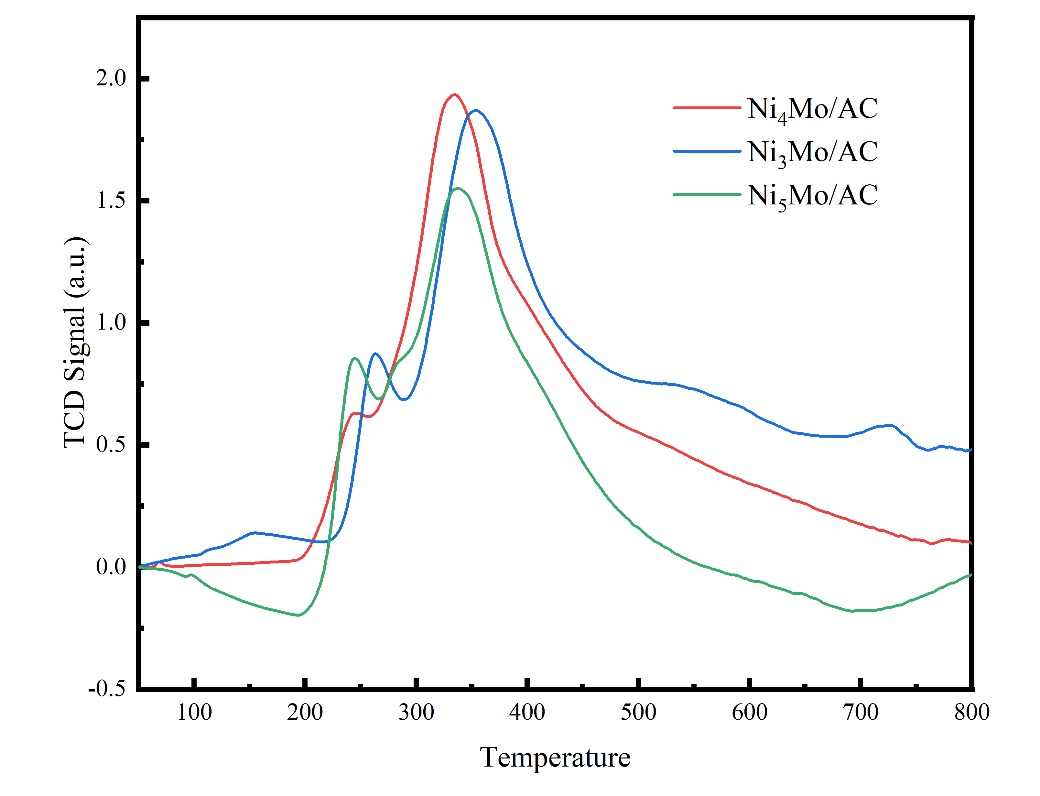


**Figure S5.** H_2_**-**TPR patterns of samples with different ratio of Ni to Mo.


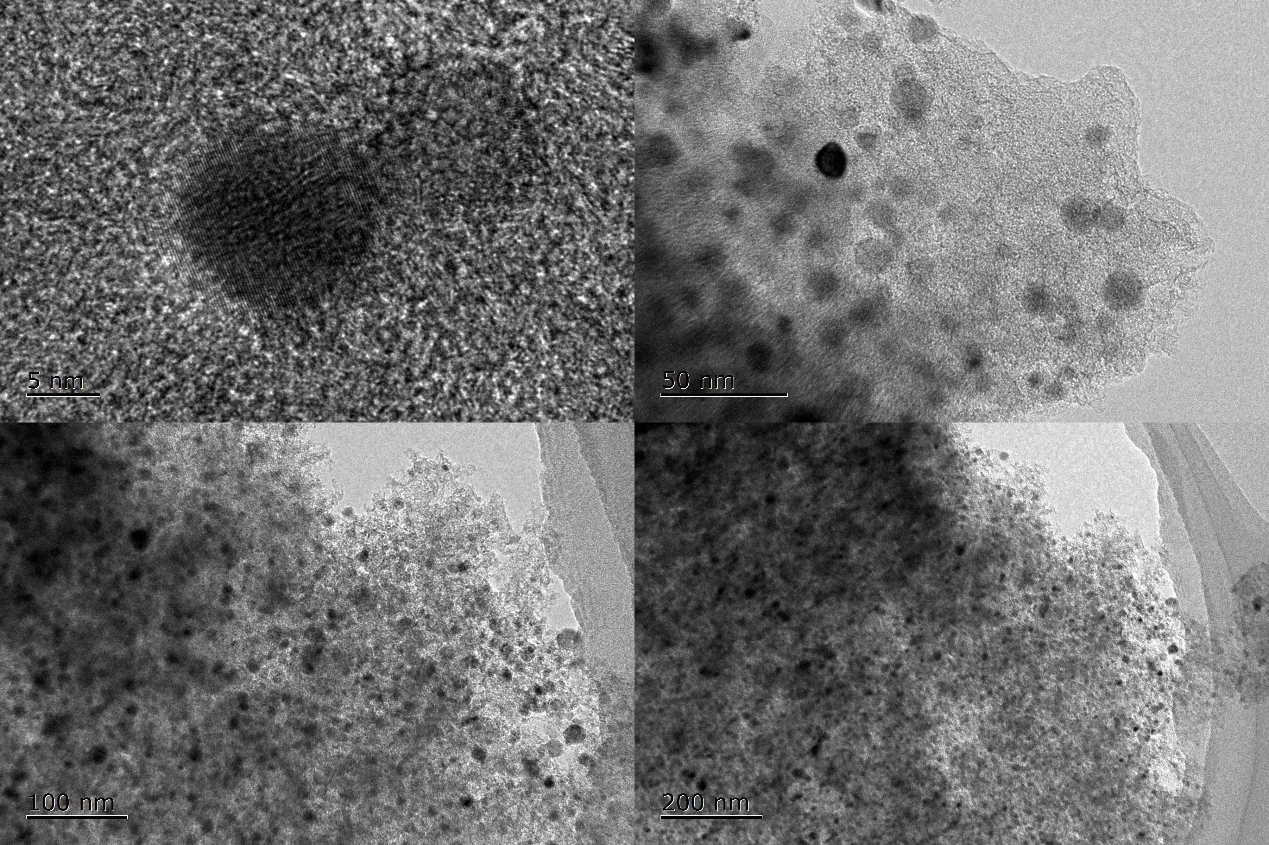


**Figure S6.** TEM images of Ni_4_Mo/AC.
